# Frequency-Guided Dynamic Hypergraph Learning for Traffic Flow Forecasting

**DOI:** 10.3390/s26165238

**Published:** 2026-08-19

**Authors:** Wanqi Li, Bin Wang, Gang Li, Yan Ma, Botao Jiang

**Affiliations:** 1 College of Information, Mechanical and Electrical Engineering, Shanghai Normal University, Shanghai 201418, China; shu_liwq@163.com (W.L.); ma-yan@shnu.edu.cn (Y.M.); 2 Zhongke Zidong Information Technology (Beijing) Co., Ltd., Beijing 100190, China; ligang@people-ai.cn; 3 China Satellite Network Application Co., Ltd., Beijing 100000, China

**Keywords:** traffic flow forecasting, spatio-temporal graph neural networks, dynamic hypergraph learning, frequency decomposition

## Abstract

Accurate traffic flow forecasting requires modeling both stable macroscopic dependencies and abrupt local fluctuations in complex road networks. Existing spatiotemporal forecasting models usually learn spatial structures from raw time-domain traffic signals, where low-frequency trends and high-frequency fluctuations are entangled. Although decomposition-based and frequency-aware methods have shown the benefit of separating heterogeneous traffic components, how frequency decomposition can support reliable high-order topology learning remains less explored. To address this issue, we propose FEDHNet, a Frequency-Guided Dynamic Hypergraph Network for traffic flow forecasting. FEDHNet first performs adaptive spectral decomposition on the hidden representation to obtain low-frequency and complementary high-frequency latent components. The low-frequency branch constructs dynamic hyperedges from the relatively smooth latent representation to model non-local high-order dependencies, while the high-frequency branch employs a lightweight 2D Inception module with GLU-based gated denoising to model rapidly varying latent responses. A low-frequency-anchored residual fusion module then adaptively integrates high-frequency residual information into the low-frequency latent representation for multi-step prediction. Experiments on four public PeMS datasets show that FEDHNet achieves competitive forecasting accuracy and multi-horizon performance, together with favorable computational efficiency compared with recent spatiotemporal forecasting baselines. Further analyses examine the effects of topology-source selection and controlled high-frequency residual modeling, revealing that the benefit of low-frequency hypergraph construction is dataset-dependent.

## 1. Introduction

Accurate traffic flow forecasting is fundamental to intelligent transportation systems (ITSs) [1], with practical applications such as route planning, traffic management, and congestion alleviation [2]. However, traffic data exhibit highly nonlinear and complex spatiotemporal dynamics, which makes accurate forecasting challenging [3].

Early deep learning methods mainly treated traffic forecasting as grid-based spatial modeling [4] or one-dimensional sequential modeling [5]. These approaches have limited ability to represent the complex topology of road networks. Early work on deep traffic forecasting [6] used stacked autoencoders to capture spatiotemporal correlations. Subsequently, Spatio-Temporal Graph Neural Networks (STGNNs) [7] became a mainstream paradigm by formulating road networks as non-Euclidean graph structures. Representative models such as DCRNN [8] and STGCN [9] aggregate spatial information through predefined physical adjacencies. However, static physical topologies cannot fully capture time-varying spatial correlations [10]. Later studies introduced adaptive matrices [11] and attention mechanisms [12] to infer latent dynamic node correlations. More recently, Transformer-based architectures [13] and continuous-time models [14] have further improved the modeling of heterogeneous and long-range spatiotemporal dependencies.

**Figure 1:**
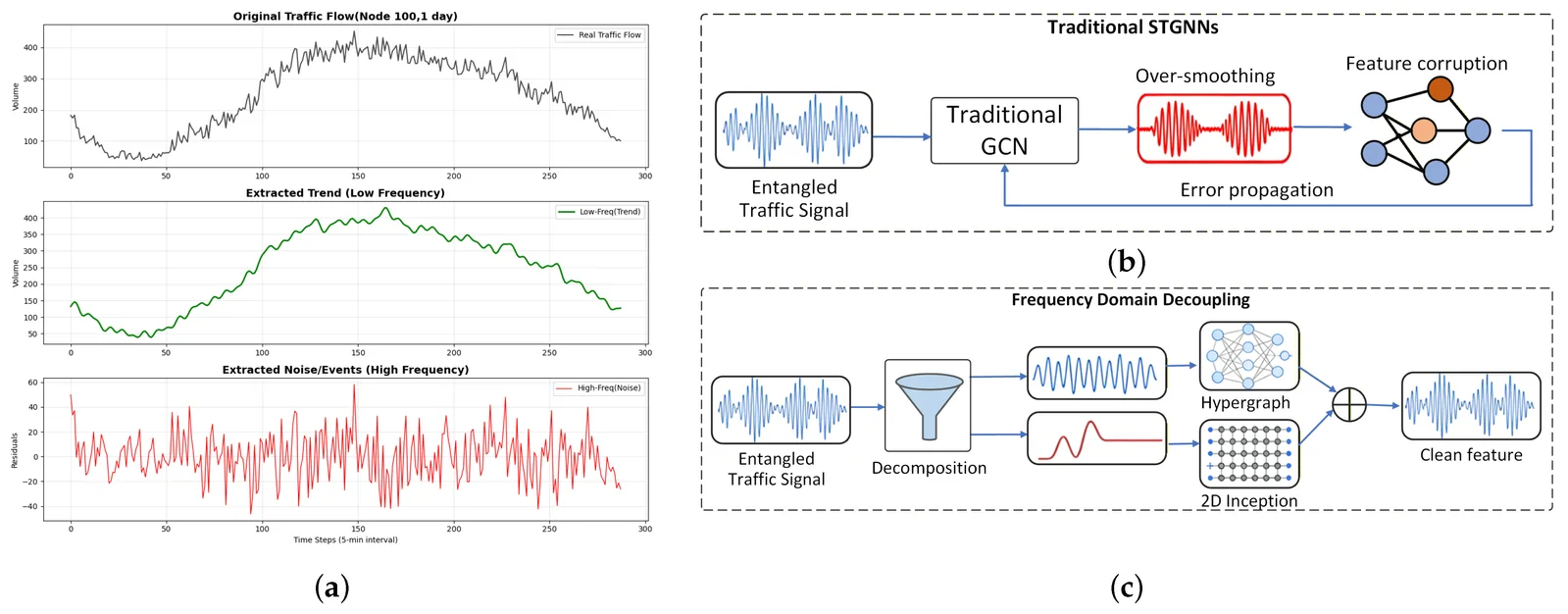
Illustration of mixed-frequency representation and frequency-guided decoupling for topology learning. (**a**) Time-domain signal and frequency decomposition; (**b**) potential feature interference in conventional topology learning; (**c**) frequency-domain decoupling.

Despite these advances, many forecasting models still learn spatial dependencies from raw traffic representations, where different temporal patterns are mixed together. As shown in Figure 1a, traffic observations may exhibit both slowly varying and rapidly varying temporal patterns. This observation motivates the frequency-domain separation used in FEDHNet. Since the decomposition in our model is applied to the learned hidden representation rather than directly to the original traffic-flow signal, the resulting low- and high-frequency components are interpreted as relatively slowly and rapidly varying latent features, respectively, rather than as direct physical proxies for specific traffic phenomena. Learning topologies from such mixed representations can be problematic. In conventional graph-based models, node representations are updated by aggregating information from connected or highly correlated nodes. When transient fluctuations are directly involved in topology learning, the model may assign strong connections to nodes that only behave similarly within a short time interval. This effect is illustrated in Figure 1b: Localized fluctuations may propagate through the learned structure and disturb the representations of other nodes. The problem becomes more pronounced in hypergraph learning, where each hyperedge connects multiple nodes, and a noisy hyperedge may affect a group of sensors simultaneously. This observation motivates the design in Figure 1c. Instead of constructing hyperedges from raw entangled representations, we first separate traffic representations in the frequency domain. The low-frequency latent representation is used to learn dynamic hyperedges, providing a smoother representation basis for high-order spatial aggregation. The high-frequency component is kept out of hyperedge construction and is processed by a separate local branch to preserve useful abrupt variations while suppressing unreliable noise responses.

Recent studies have attempted to reduce the interference among heterogeneous traffic patterns through decomposition-based or frequency-aware modeling. For example, seasonal-trend decomposition and Fourier-enhanced representations have been used to capture global temporal properties in long-term forecasting [15]. Traffic-specific models further separate traffic signals into periodic and perturbative components and process them with dual-branch architectures [16]. DEC-Former and STDN also show that decomposition can improve attention mechanisms, spatiotemporal embeddings, and dynamic graph learning [17,18]. These studies confirm the value of separating heterogeneous traffic dynamics. However, most of them use the separated components to improve temporal attention, recurrent modeling, graph convolution, or component-wise feature extraction. How frequency decomposition can support reliable high-order topology learning remains less explored. This issue is particularly important in hypergraph-based forecasting because a noisy hyperedge can influence multiple sensors at once. Therefore, directly constructing hyperedges from raw entangled signals may lead to unreliable high-order spatial dependencies.

Motivated by these observations, we propose a Frequency-Guided Dynamic Hypergraph Network, termed FEDHNet. The central design of FEDHNet is to couple frequency decomposition with dynamic high-order topology learning. Specifically, adaptive spectral decomposition is used to obtain frequency-specific latent representations, and the low-frequency latent representation is employed to guide dynamic hypergraph construction. This design reduces the direct influence of rapidly varying latent components on hyperedge generation and provides a smoother representation basis for high-order topology inference. The complementary high-frequency latent representation is processed by a lightweight 2D Inception module with GLU-based gated denoising, and a low-frequency-anchored residual fusion module integrates the two branches for multi-step forecasting. The main contributions are summarized as follows:

- We develop a frequency-guided dynamic hypergraph construction strategy that couples spectral decomposition with high-order topology learning. Instead of constructing dynamic hyperedges directly from mixed latent representations, FEDHNet uses the low-frequency latent representation as the topology-learning input, reducing the direct influence of rapidly varying components on hyperedge generation.
- We propose a frequency-specific dual-branch architecture. The low-frequency branch employs dynamic hypergraph learning to capture non-local high-order dependencies, while the high-frequency branch uses 2D Inception-based gated modeling to extract complementary rapidly varying features.
- We design a low-frequency-anchored residual fusion mechanism, where high-frequency features are treated as adaptive residual corrections to the low-frequency latent representation for multi-step prediction.
- Experiments on four public PeMS datasets demonstrate that FEDHNet achieves competitive forecasting accuracy and multi-horizon performance and favorable computational efficiency. Additional ablation and visualization analyses further examine the role of frequency-guided hypergraph construction.

## 2. Related Work

### 2.1. Spatiotemporal Graph Neural Networks

Graph neural networks (GNNs) have advanced traffic forecasting by explicitly modeling network topologies. Early models such as DCRNN [8] and STGCN [9] combine graph convolutions with temporal modules, but they rely heavily on predefined static graphs. To capture time-varying traffic patterns, later studies introduced adaptive mechanisms. Graph WaveNet [19] and AGCRN [11] infer implicit spatial dependencies through adaptive adjacency matrices and node-specific parameter learning. DGCN-RL [20] employs reinforcement learning for dynamic graph generation. Other studies, such as STSGCN [21] and ASTGNN [22], explore fine-grained spatiotemporal interaction modeling. Recent studies have further investigated more flexible forms of spatiotemporal dependency learning. STPGNN [23] identifies pivotal nodes with strong traffic aggregation and distribution characteristics and constructs a pivotal graph to model the complex spatiotemporal dependencies surrounding these nodes. Transformer-based architectures, including PDFormer [2], STAEformer [13], DTRformer [24], and SSL-STMFormer [25], further improve the modeling of complex spatiotemporal dependencies. In particular, SSL-STMFormer combines spatial and temporal attention with entanglement-aware modules and self-supervised learning to capture dynamic long-range dependencies and spatiotemporal heterogeneity. More recently, MetaDG [26] extends dynamic graph modeling by generating dynamic node representations, adjacency matrices, and meta-parameters at each time step while further refining message propagation through dynamic graph qualification. Despite these advances, existing methods mainly focus on improving pairwise graph structures, attention mechanisms, dynamic adjacency learning, or spatiotemporal representation modeling. The influence of frequency-specific latent information on high-order topology construction remains less explored. Different from conventional pairwise graph models, FEDHNet couples frequency decomposition with dynamic hypergraph learning and uses a relatively low-frequency latent representation to guide high-order topology inference.

### 2.2. Decomposition-Based and Frequency-Aware Modeling

Decomposition-based methods aim to separate heterogeneous temporal patterns before prediction. Autoformer [27] introduces seasonal-trend decomposition into Transformer-based forecasting, while FEDformer [15] combines decomposition with frequency-enhanced representations to capture global properties of time series. In traffic forecasting, DEC-Former [17] decomposes traffic series into trend and seasonal components and repositions attention mechanisms to more suitable temporal and spatial contexts. STDN [18] incorporates spatiotemporal embeddings into trend-seasonality decomposition and dynamic relationship graph learning. These studies show that decomposed representations can reduce interference among heterogeneous traffic patterns. Frequency-aware traffic forecasting methods further exploit spectral representations to distinguish information distributed across different frequency bands. FEDDGCN [16] integrates Fourier filters with a gated decoupling mechanism for frequency-aware traffic representation learning. These recent studies demonstrate the growing interest in decomposition- and frequency-aware modeling for traffic forecasting. However, decomposition is mainly employed to improve temporal attention, graph convolution, recurrent modeling, or component-wise feature extraction. In contrast, FEDHNet further couples frequency decomposition with dynamic high-order topology learning, using the relatively low-frequency latent representation as the input for hyperedge construction.

### 2.3. High-Order Topology and Hypergraph Learning

Conventional GNNs are limited to pairwise connections and may fail to capture non-local and multi-way interactions in complex urban road networks. Recent hypergraph-based models have been explored for passenger flow, traffic flow, and traffic speed forecasting [28,29,30,31,32]. DHCN [28] introduces dynamic hypergraph convolution for metro passenger flow prediction, while DyHSL [29] learns dynamic hypergraph structures for traffic flow forecasting. ST-HCN [31] and DSTHGCN [30] integrate spatiotemporal hypergraph modeling into traffic forecasting, and MvHSTM [32] explores multi-view hypergraph modeling for traffic speed forecasting. Most existing hypergraph-based forecasting methods infer hyperedge structures directly from raw or mixed traffic representations. Rapid variations contained in these representations may affect hyperedge assignments and consequently introduce fluctuations into high-order topology learning. FEDHNet instead performs dynamic hypergraph construction on the relatively low-frequency latent representation, providing a smoother representation basis for estimating hyperedge memberships. The complementary high-frequency latent representation is processed through a separate 2D Inception-based branch for local feature modeling, thereby preventing rapidly varying components from directly participating in hyperedge generation.

## 3. Problem Formulation

Following the standard paradigm in spatiotemporal forecasting [8,9], we formulate a traffic network as a directed graph $G=(V,E,A_{\mathrm{pre}})$, where V denotes the set of *N* traffic sensors, E denotes the set of road-network edges, and $A_{\mathrm{pre}}\in R^{N\times N}$ is the predefined proximity-based adjacency matrix. Let $X_{t}\in R^{N\times C}$ denote the graph signal observed at time step *t*, where *C* is the number of node features. Given a historical observation tensor $X=\left[X_{t-T+1},\ldots ,X_{t}\right]\in R^{T\times N\times C}$, our objective is to learn a mapping function F(·) that predicts the future traffic conditions $\hat{Y}=\left[{\hat{X}}_{t+1},\ldots ,{\hat{X}}_{t+T^{\prime }}\right]$ for the next $T^{\prime }$ steps:

(1) $$\hat{Y}=F\left(X;G\right)$$

where $\hat{Y}\in R^{T^{\prime }\times N\times C_{\mathrm{out}}}$ represents the predicted graph signals, with $C_{\mathrm{out}}$ being the output feature dimension.

## 4. Methodology

**Figure 2:**
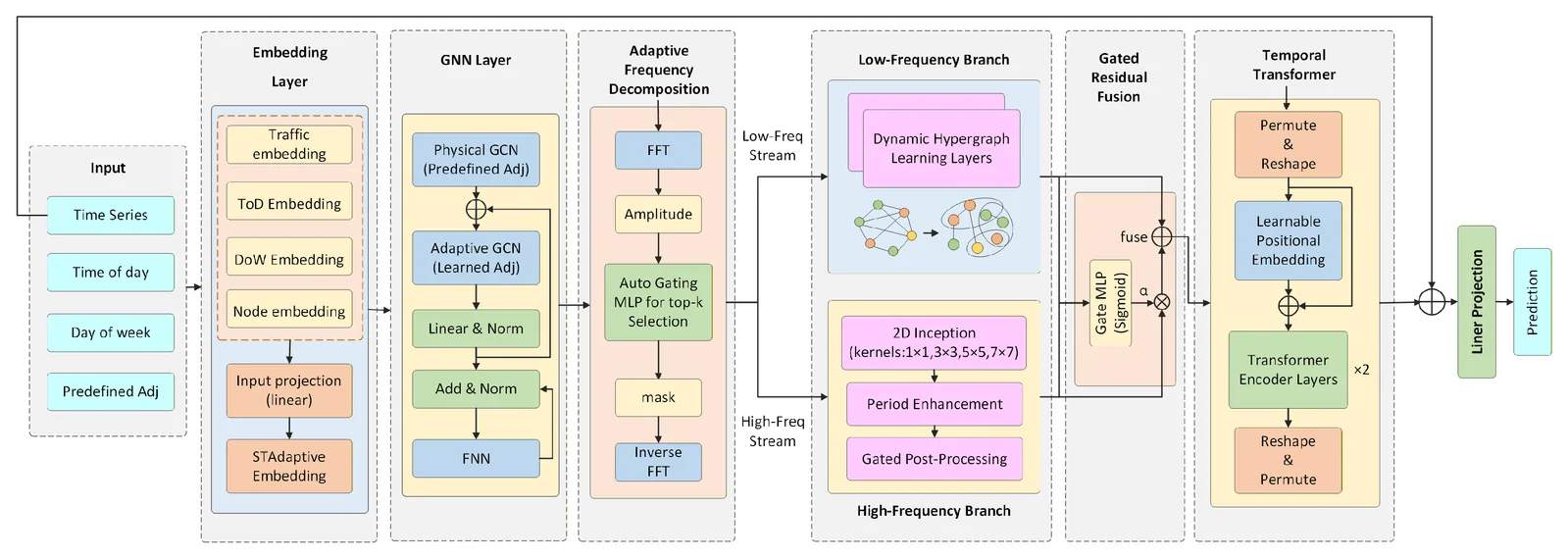
The overall architecture of the proposed FEDHNet.

The overall architecture of the proposed Frequency-Guided Dynamic Hypergraph Network (FEDHNet) is illustrated in Figure 2. The model first embeds raw traffic sequences with temporal and spatial adaptive embeddings and then applies a graph convolution layer to obtain structurally smoothed representations. An adaptive spectral decomposition module separates the hidden representation into low-frequency and complementary high-frequency latent components. The low-frequency branch learns dynamic hyperedges from the relatively smooth latent representation to model non-local high-order dependencies. The high-frequency branch uses a multi-scale 2D Inception module with GLU-based gated denoising to model rapidly varying latent responses. Finally, a low-frequency-anchored residual fusion module integrates the two branches for RevIN-based multi-step prediction.

### 4.1. Spatiotemporal Embedding and Adaptive Graph Construction

We first map the input tensor $X\in R^{T\times N\times C}$ into a high-dimensional continuous feature space. To encode periodic temporal contexts, discrete time-of-day (TOD) and day-of-week (DOW) indices are mapped into vector representations [9,13]. These temporal embeddings are concatenated with X along the feature dimension and projected to a hidden dimension *D* through a linear transformation. To capture spatiotemporal heterogeneity across the road network, a learnable spatiotemporal adaptive embedding tensor $E_{\mathrm{st}}\in R^{T\times N\times D}$ is added to the projected features [11,13], yielding the initialized representation $X_{\mathrm{emb}}\in R^{T\times N\times D}$.

Next, a preliminary GNN layer performs spatial structural smoothing on $X_{\mathrm{emb}}$. Let $Z=X_{\mathrm{emb}}W_{\mathrm{in}}\in R^{T\times N\times D}$ denote the hidden state after linear projection. Relying only on the predefined physical adjacency matrix $A_{\mathrm{pre}}$ limits the model’s ability to capture implicit non-Euclidean spatial correlations [19]. To alleviate this limitation, we construct an adaptive adjacency matrix $A_{\mathrm{adp}}$ using node-specific learnable dictionaries $E_{1},E_{2}\in R^{N\times d}$, where *d* is the embedding dimension for graph generation [11]. A ReLU activation is applied to suppress negative affinity scores before row-wise normalization:

(2) $$A_{\mathrm{adp}}=\mathrm{Softmax}\left(\mathrm{ReLU}(E_{1}E_{2}^{⊤})\right)$$

where the Softmax function normalizes the adaptive edge weights row-wise (i.e., for each node *i*, the attention scores across all nodes *j* sum to 1). The predefined physical adjacency matrix A is constructed from road-network distances following the distance-based graph construction in DCRNN [8]. Specifically, $A_{ij}=\exp\left[-{\left(d_{ij}/\sigma \right)}^{2}\right]$, and weights below ϵ=0.1 are set to zero. Self-connections are retained ($A_{ii}=1$), and row-wise random-walk normalization is applied as $A_{\mathrm{pre}}=D^{-1}A$, where $D_{ii}=\sum_{j}A_{ij}$. No additional identity matrix is added during normalization. Neighborhood information is then aggregated over the augmented graph topology to obtain the intermediate aggregated state $H_{\mathrm{agg}}\in R^{T\times N\times D}$:

(3) $$H_{\mathrm{agg}}=\left(A_{\mathrm{pre}}+A_{\mathrm{adp}}\right)Z$$

where the graph convolution is applied along the spatial dimension across all *T* time steps, computed via Einstein summation to handle the high-dimensional tensors. The spatially smoothed representation H is generated through a sequential combination of residual connections, layer normalization (LN), and a feed-forward network (FFN) [22]. To ensure clear structural formulation, this process is decoupled into two sequential steps. First, the aggregated state is added to the initial representation and normalized to stabilize the training dynamics:

(4) $$H^{\prime }=\mathrm{LN}\left(H_{\mathrm{agg}}+Z\right)$$

Subsequently, the normalized representation $H^{\prime }$ is processed by the FFN and refined via a second residual connection to produce the final output:

(5) $$H=H^{\prime }+\mathrm{FFN}\left(H^{\prime }\right)$$

This spatial smoothing stage ensures that the subsequent spectral decomposition operates on representations with refined structural dependencies.

### 4.2. Adaptive Spectral Decomposition

The spectral decomposition in FEDHNet is performed on the hidden representation H. Therefore, the resulting frequency components are interpreted in the latent feature space instead of being assigned explicit physical meanings. The low-frequency latent representation emphasizes relatively slowly varying components of H, whereas the complementary high-frequency latent representation retains more rapidly varying information, which may contain both useful transient responses and representation noise. FEDHNet uses the low-frequency latent representation for dynamic hypergraph learning and preserves the high-frequency latent representation for local variation modeling.

**Figure 3:**
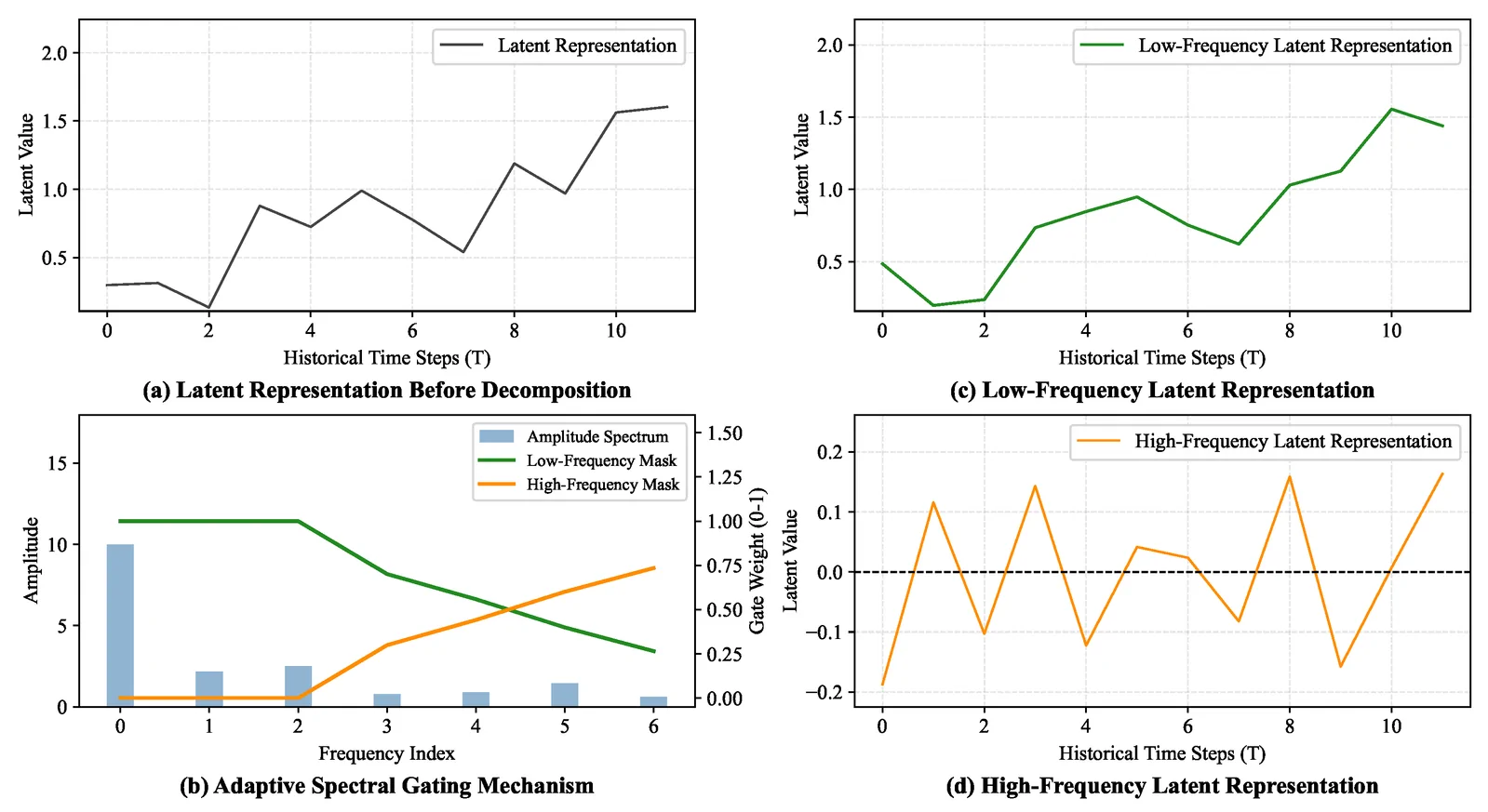
Illustration of the adaptive spectral decomposition module. The dashed horizontal line in (**d**) indicates the zero baseline for the high-frequency latent representation.

Different from time-domain seasonal-trend decomposition methods [27], this module modulates representations directly in the spectral domain, as shown in Figure 3. Given the real-valued nature of traffic sequences, we apply the real fast Fourier transform (RFFT) along the temporal dimension of the hidden representation $H\in R^{T\times N\times D}$. This operation removes conjugate redundancy and reduces computational overhead, yielding the complex frequency spectrum $\hat{H}\in C^{F\times N\times D}$, where F=⌊T/2⌋+1. The corresponding amplitude spectrum is denoted as $A=|\hat{H}|$.

To adaptively determine the frequency modulation weights for varying traffic conditions, a multi-layer perceptron (MLP) takes the amplitude A as input to yield a learnable soft mask $M_{\mathrm{soft}}\in R^{F\times N\times D}$, integrated with a monotonically decreasing bias vector $b_{\mathrm{init}}\in R^{F}$:

(6) $$M_{\mathrm{soft}}=\sigma \left(\mathrm{MLP}\left(A\right)+b_{\mathrm{init}}\right)$$

where σ(·) denotes the Sigmoid activation function, mapping the output to (0, 1). This mechanism serves as a learnable soft filter that adaptively determines the retention weight of each frequency band rather than relying on a fixed frequency cutoff. The bias $b_{\mathrm{init}}$ introduces an inductive preference toward lower-frequency components by assigning them higher initial retention probabilities, while the final spectral weights remain adaptively determined from the latent representation.

To avoid over-suppressing essential low-frequency information, a prior hard mask $M_{\mathrm{hard}}$ is introduced to preserve the direct current (DC) component and several fundamental low-frequency bands. $M_{\mathrm{hard}}$ is defined as a binary step function based on the frequency index f∈{0,1,…,F−1}:

(7) $$M_{\mathrm{hard}}\left(f\right)=\left\{\begin{array}{cc} 1, & \mathrm{if}\;f\leq f_{c}\\ 0, & \mathrm{otherwise} \end{array}\right.$$

where $f_{c}$ is the cutoff frequency index. In our implementation, $f_{c}$ is set to 2, preserving the DC component and the first two low-frequency bands within the observation window. This design ensures the preservation of the lowest-frequency information while allowing the remaining frequency bands to be adaptively modulated. The complementary masks for the low- and high-frequency components are then formulated as follows:

(8) $$M_{\mathrm{low}}=\max\left(M_{\mathrm{soft}},M_{\mathrm{hard}}\right),\;M_{\mathrm{high}}=1-M_{\mathrm{low}}$$

The decoupled representations are reconstructed back into the time domain via the Inverse RFFT (IRFFT) utilizing the Hadamard product (⊙) between the complex spectrum $\hat{H}$ and the respective masks, yielding the low-frequency latent representation $X_{\mathrm{low}}\in R^{T\times N\times D}$ and the complementary high-frequency latent representation $X_{\mathrm{high}}\in R^{T\times N\times D}$:

(9) $$X_{\mathrm{low}}=\mathrm{IRFFT}\left(\hat{H}⊙M_{\mathrm{low}}\right),\;X_{\mathrm{high}}=\mathrm{IRFFT}\left(\hat{H}⊙M_{\mathrm{high}}\right)$$

### 4.3. Low-Frequency Branch: Dynamic Hypergraph Learning

The decoupled low-frequency tensor $X_{\mathrm{low}}\in R^{T\times N\times D}$ is regarded as a relatively smooth representation in the latent feature space. This property is particularly relevant to dynamic hypergraph construction because the hyperedge memberships are directly inferred from $X_{\mathrm{low}}$. As formulated below, the incidence tensor is generated through a learnable projection followed by Softmax, i.e., $H_{\mathrm{inc}}=\mathrm{Softmax}\left(X_{\mathrm{low}}W_{\mathrm{struct}}\right)$. Therefore, rapidly varying components in the input representation can directly affect the hyperedge-assignment logits and consequently introduce fluctuations into the inferred topology. The adaptive spectral decomposition attenuates part of the rapidly varying information before hyperedge generation so that the representation used for topology inference varies more smoothly along the temporal dimension. Consequently, the hyperedge memberships inferred from $X_{\mathrm{low}}$ are expected to be less directly affected by short-term variations than those inferred from the mixed representation before decomposition. This provides the rationale for using the low-frequency latent representation as the input to dynamic hypergraph construction. Importantly, this interpretation does not assume that the low-frequency representation exclusively corresponds to specific physical traffic patterns, nor does it imply a theoretical guarantee of superior topology learning. From a traffic perspective, geographically separated functional zones may exhibit synchronized peak-hour behavior even when they are not directly connected by roads. Standard pairwise graph convolutional networks (GCNs) [9,33] are structurally limited in modeling such high-order semantic dependencies because they mainly capture pairwise relationships defined by physical adjacency or simple similarity. Inspired by hypergraph neural networks for traffic forecasting [28,30,31,32] and dynamic structure learning paradigms [34,35], we introduce a dynamic hypergraph learning module.

**Figure 4:**
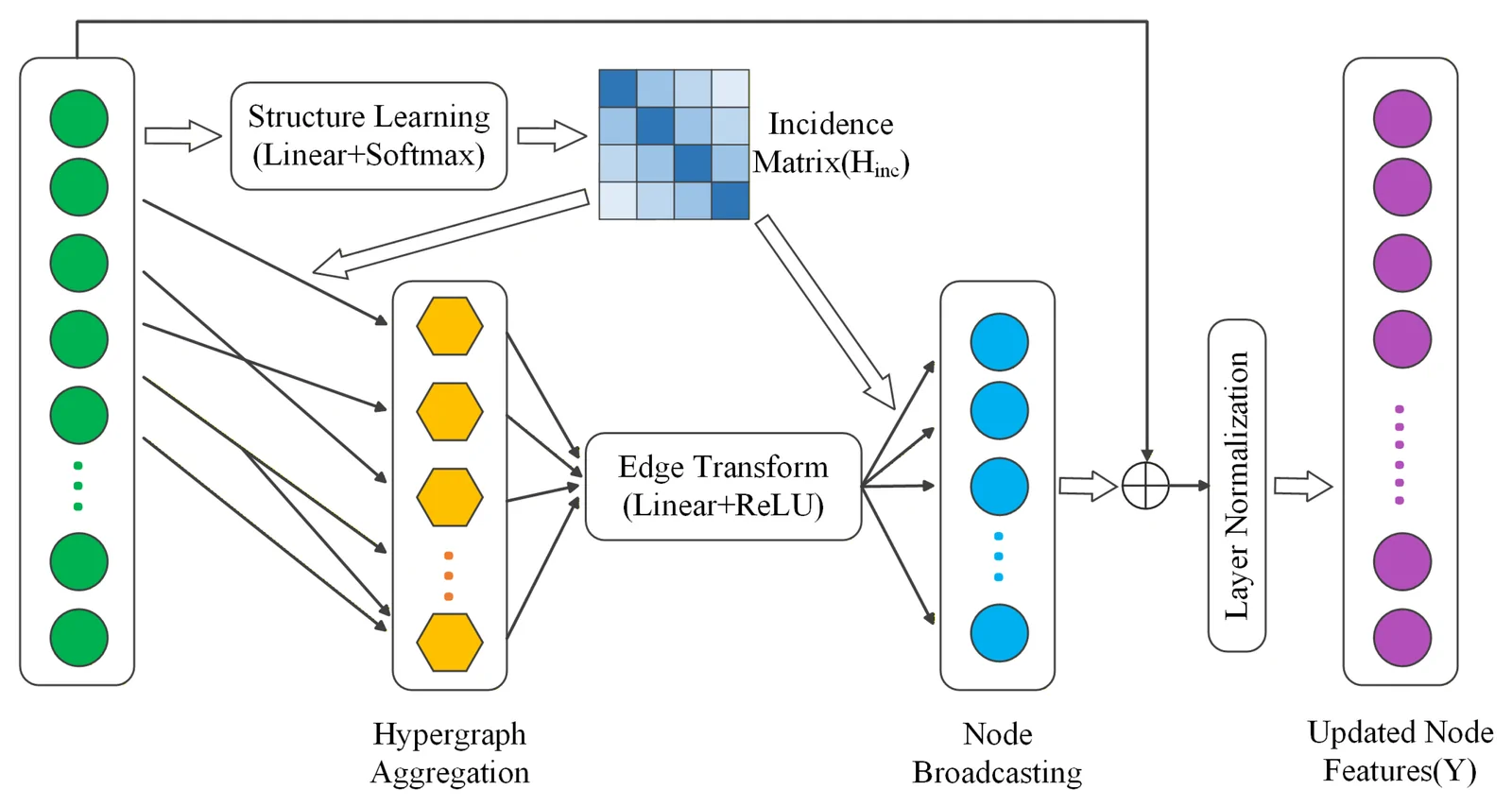
The message-passing paradigm of the dynamic hypergraph learning module.

As illustrated in Figure 4, the learning paradigm initiates with a dynamic structure learning phase. Unlike methods that rely on predefined hypergraphs based on spatial proximity or static semantic rules [31,32], our approach maps the *N* physical nodes into *K* latent semantic hyperedges in a data-driven manner without predefined hypergraph incidence priors. A dynamic incidence tensor $H_{\mathrm{inc}}\in R^{T\times N\times K}$ is learned directly from the decoupled low-frequency features $X_{\mathrm{low}}$:

(10) $$H_{\mathrm{inc}}=\mathrm{Softmax}\left(X_{\mathrm{low}}W_{\mathrm{struct}}\right)$$

where $W_{\mathrm{struct}}\in R^{D\times K}$ is a learnable projection matrix. The Softmax function is applied along the spatial dimension across the *N* nodes. This operation enforces that the membership weights of all physical nodes within any generated latent hyperedge sum to 1, thereby identifying the relative importance of distinct traffic sensors within the same learned latent hyperedge.

The message-passing protocol on the generated hypergraph is formalized through aggregation and broadcasting [34]. Firstly, in the Hypergraph Aggregation step, node features are projected to formulate the hyperedge representations. At each time step *t*, the hyperedge matrix $U_{t}\in R^{K\times D}$ is computed as follows:

(11) $$U_{t}=H_{\mathrm{inc},t}^{⊤}X_{\mathrm{low},t}$$

where ^⊤^ denotes the transpose over the spatial and hyperedge dimensions. Subsequently, an edge transform operation refines the hyperedge semantics via non-linear activation. The updated representations are propagated back to the constituent nodes via node broadcasting. A residual connection followed by layer normalization (LN) [22] yields the refined node features $X_{\mathrm{low}}^{\left(\mathrm{out}\right)}\in R^{T\times N\times D}$:

(12) $$X_{\mathrm{low}}^{\left(\mathrm{out}\right)}=\mathrm{LN}\left(X_{\mathrm{low}}+H_{\mathrm{inc}}\mathrm{ReLU}\left(UW_{\mathrm{edge}}\right)\right)$$

where $W_{\mathrm{edge}}\in R^{D\times D}$ is the hyperedge transformation matrix. Unlike conventional degree-normalized hypergraph convolution, the above propagation directly performs node-to-hyperedge aggregation and hyperedge-to-node broadcasting without explicitly introducing node- and hyperedge-degree normalization. We further compare this simplified propagation with a conventional normalized variant in Section 5.5. This paradigm enables the low-frequency branch to capture non-local high-order dependencies from a relatively smooth latent representation that may transcend physical spatial proximity, addressing the structural limitation of conventional pairwise graph models [9].

### 4.4. High-Frequency Branch: 2D Inception and Gated Denoising

The decoupled high-frequency tensor $X_{\mathrm{high}}\in R^{T\times N\times D}$ emphasizes relatively rapidly varying responses in the latent feature space. These responses may encode useful transient information and representation noise. Therefore, rather than using them directly for hyperedge construction, we route them to a specialized branch for complementary local feature modeling. Consequently, we integrate the predefined physical adjacency matrix $A_{\mathrm{pre}}$ via a standard graph convolution [9,33]:

(13) $$X_{\mathrm{sp}}=X_{\mathrm{high}}+\mathrm{LN}\left(\mathrm{ReLU}\left(A_{\mathrm{pre}}X_{\mathrm{high}}W_{\mathrm{sp}}\right)\right)$$

where $W_{\mathrm{sp}}\in R^{D\times D}$ is the learnable spatial projection weight.

Standard 1D convolutions may require deeper architectures or enlarged receptive fields to jointly model short-term variations and cross-cycle temporal dependencies. To overcome this, we introduce a temporal-to-spatial mapping mechanism that projects implicit temporal periodicity into explicit 2D spatial receptive fields. This process begins with a data-driven dominant period discovery mechanism [36]. Operationally, FFT is applied along the temporal dimension of each sensor sequence in $X_{\mathrm{high}}$. After removing the zero-frequency (DC) component, the frequency index with the maximum amplitude is identified for each sensor sequence in the current mini-batch. To reduce unstable sequence-level estimates, mode voting is then performed over these dominant-frequency indices, yielding a batch-level dominant frequency $f_{\mathrm{mode}}$. The folding period is determined as $P=\max(2,\left\lfloor T/f_{\mathrm{mode}}\right\rfloor )$. Compared with methods relying on fixed periodic assumptions [12,37], this mechanism determines the folding period from the current latent representation. The voting operation is theoretically dependent on batch composition; however, the discrete frequency-to-period mapping allows neighboring frequency indices to produce the same folding period. For example, when T=12, both $f_{\mathrm{mode}}=5$ and $f_{\mathrm{mode}}=6$ yield P=2. If no valid dominant frequency is detected, *P* defaults to the full window length *T*. We further evaluate the practical sensitivity of this mechanism to inference batch size in Section 5.11.

Given the dynamically discovered period *P*, the input sequence $X_{\mathrm{sp}}\in R^{T\times N\times D}$ is zero-padded along the temporal dimension to $T_{\mathrm{pad}}$ (the nearest multiple of *P*), yielding the padded representation $X_{\mathrm{pad}}$. We define a Fold(·) operation to map this 1D sequence into a 2D grid tensor $X_{\mathrm{grid}}\in R^{S\times P\times N\times D}$, where $S=T_{\mathrm{pad}}/P$ represents the number of periodic cycles. This mapping is formulated as

(14) $$X_{\mathrm{grid}}\left(s,p,n,d\right)=X_{\mathrm{pad}}\left(s\cdot P+p,n,d\right)$$

where s∈{0,1,…,S−1} indexes the inter-period cycle, and p∈{0,1,…,P−1} denotes the intra-period phase.

This transformation effectively aligns periodic traffic fluctuations across different cycles into identical spatial columns. By applying multi-scale 2D Inception blocks (using 1×1, 3×3, 5×5, and 7×7 kernels) [38,39] to $X_{\mathrm{grid}}$, the network extracts both short-term intra-period continuity along the phase axis (*p*) and long-range inter-period recurrence along the cycle axis (*s*) under varying receptive fields.

After 2D feature extraction, an inverse mapping Unfold(·) restores the tensor to the original 1D temporal space and truncates the padded elements. A gated linear unit (GLU) [40] is then applied to modulate high-frequency responses. The reshaped feature map is projected and partitioned along the channel dimension into candidate burst features V and a controlling gate G:

(15) $$\left[V\|G\right]=\mathrm{Unfold}\left({\mathrm{Inception}}_{2D}\left(X_{\mathrm{grid}}\right)\right)W_{\mathrm{gate}}$$

(16) $$X_{\mathrm{high}}^{\left(\mathrm{out}\right)}=V⊙\sigma \left(G\right)$$

where $W_{\mathrm{gate}}\in R^{D_{\mathrm{inc}}\times 2D}$ is the projection weight matrix, $D_{\mathrm{inc}}$ denotes the output channel dimension of the Inception block, ‖ represents the concatenation operator, and ⊙ is the Hadamard product. The gate σ(G) adaptively modulates rapidly varying responses and can attenuate potentially noisy components.

### 4.5. Residual Gated Fusion and Prediction

To fuse the heterogeneous features from the two branches, we adopt a low-frequency-anchored residual fusion module. Since the low-frequency branch provides topology-aware high-order representations, its output serves as the primary representation, while the high-frequency branch provides complementary residual information for rapidly varying patterns. A fusion gate $G_{\mathrm{fuse}}$ adaptively modulates the high-frequency feature $X_{\mathrm{high}}^{\left(\mathrm{out}\right)}$ before adding it to the low-frequency feature $X_{\mathrm{low}}^{\left(\mathrm{out}\right)}$. Similarly to gated linear units [40], this mechanism controls the residual information flow as follows:

(17) $$G_{\mathrm{fuse}}=\sigma \left(\left[X_{\mathrm{low}}^{\left(\mathrm{out}\right)}\|X_{\mathrm{high}}^{\left(\mathrm{out}\right)}\right]W_{\mathrm{fuse}}\right)$$

(18) $$X_{\mathrm{fused}}=X_{\mathrm{low}}^{\left(\mathrm{out}\right)}+G_{\mathrm{fuse}}⊙X_{\mathrm{high}}^{\left(\mathrm{out}\right)}$$

where $W_{\mathrm{fuse}}\in R^{2D\times D}$ is the learnable weight matrix for the fusion gate, and ‖ denotes concatenation along the feature dimension. The fused representation $X_{\mathrm{fused}}$ is fed into a temporal Transformer to model long-range sequential dependencies [13]. To mitigate distribution shifts in time-series forecasting, the final representation is mapped through a dense MLP layer and denormalized using the statistics recorded during reversible instance normalization (RevIN) [41]. This step restores the original data scale and produces the final multi-step prediction tensor $\hat{Y}\in R^{T^{\prime }\times N\times C_{\mathrm{out}}}$.

## 5. Experiments

### 5.1. Datasets

We evaluate FEDHNet on four public real-world traffic datasets: PEMS03, PEMS04, PEMS07, and PEMS08 [21]. These datasets are collected by the Caltrans Performance Measurement System (PeMS) [42] from highway sensor networks in California, USA. The raw traffic records are aggregated into 5 min intervals, resulting in 288 data points per day. PEMS03 and PEMS07 contain traffic flow data, whereas PEMS04 and PEMS08 include three traffic measurements: total flow, average speed, and average occupancy. In our experiments, traffic flow is used as the prediction target, while the additional measurements in PEMS04 and PEMS08 are used as auxiliary input features. Table 1 summarizes the dataset statistics.

### 5.2. Baselines

We compare FEDHNet with representative forecasting baselines from four groups:

- **Classical Statistical Model:** ARIMA [43], a widely used time-series forecasting model.
- **Spatiotemporal Graph and Hypergraph Neural Networks:** DCRNN [8], STGCN [9], ASTGCN [12], Graph WaveNet [19], STSGCN [21], Z-GCNETs [44], and DyHSL [29]. DyHSL learns dynamic hypergraph structures to capture high-order spatiotemporal dependencies.
- **Continuous-Time Neural Networks:** STGODE [45] and STG-NCDE [14].
- **Advanced Spatiotemporal Forecasting Models:** STDN [18], PDFormer [2], STAEformer [13], STPGNN [23], and DTRformer [24]. Among them, STDN integrates trend-seasonality decomposition with dynamic relationship graph learning.

### 5.3. Experimental Settings

**Data Preprocessing.** Following common practice [2], we split all datasets into training, validation, and testing sets with a ratio of 6:2:2. A historical window of 12 consecutive time steps is used to predict traffic flow for the next 12 steps. To prevent data leakage, the statistical mean and variance are computed only from the training set. RevIN [41] is applied to the historical traffic-flow input channel before spatiotemporal embedding, and the corresponding instance statistics are used to restore the original scale of the predicted outputs.

**Implementation.** All experiments were conducted on a Linux server equipped with a single NVIDIA RTX 5090 GPU (NVIDIA Corporation, Santa Clara, CA, USA) using PyTorch (v2.8.0) and CUDA 12.8. We train FEDHNet with the Adam optimizer, a batch size of 64, an initial learning rate of $10^{-3}$, and a weight decay of $10^{-4}$. A cosine annealing scheduler decays the learning rate to a minimum of $10^{-6}$. The maximum number of training epochs is 300, and early stopping is applied with a patience of 30 epochs based on the validation loss. The model is optimized using Smooth L1 Loss with β=1.0. Model selection and hyperparameter tuning are based on validation performance, while the test set is used only for final evaluation. We set D=128, L=2, and K=16 for PEMS03, PEMS04, and PEMS07, while D=96, L=2, and K=8 are used for PEMS08. All reproduced baselines use the same data preprocessing, dataset split, input setting, and evaluation protocol as FEDHNet to ensure a fair comparison. For reproduced experiments, the random seed is fixed. For baselines with reported results under identical dataset splits and evaluation settings, we use their official results. Otherwise, we reproduce the models using the released source codes and tune hyperparameters according to the recommended configurations.

**Evaluation Metrics.** We evaluate prediction accuracy using the mean absolute error (MAE), root mean square error (RMSE), and mean absolute percentage error (MAPE). Following standard practice, missing and zero values are masked during evaluation.

### 5.4. Performance Comparison

Table 2 reports the mean forecasting performance over all 12 prediction horizons on the four PeMS datasets. FEDHNet consistently improves over traditional statistical models and early STGNNs, such as ARIMA, DCRNN, and STGCN, while remaining competitive with recent Transformer-based approaches. DTRformer achieves the lowest absolute errors on several datasets, whereas FEDHNet remains close to the best-performing results, particularly on PEMS07 and PEMS08. More importantly, FEDHNet shows favorable performance against the two closely related baselines. Compared with the reproduced DyHSL, which directly learns dynamic hypergraph structures without frequency-guided decomposition, FEDHNet obtains lower errors on 11 of the 12 evaluated metrics. Compared with STDN, which combines decomposition with dynamic graph learning but does not employ high-order hypergraph topology, FEDHNet achieves lower errors on 10 of the 12 metrics. These results suggest that coupling decomposition with dynamic high-order topology learning provides complementary empirical benefits beyond either dynamic hypergraph modeling or decomposition-based dynamic graph learning alone. Although FEDHNet does not uniformly achieve the lowest absolute error across all datasets and metrics, it provides a competitive overall accuracy–efficiency trade-off. The computational results in Section 5.12 further show that FEDHNet requires substantially less training and inference time than the compared Transformer-based baselines.

### 5.5. Ablation Study

We conduct two groups of ablation experiments based on the average forecasting performance over all 12 prediction horizons. The first evaluates the contribution of major components, including frequency decomposition, adaptive spectral gating, dynamic hypergraph learning, high-frequency Inception modeling, gated denoising, and adaptive spatial representation learning. The second focuses on the hypergraph’s design from two aspects: the representation used for dynamic hyperedge generation and the hypergraph propagation scheme. To further assess whether marginal differences in topology-source selection are robust to random initialization, we additionally repeat the Raw-HG and Low-HG variants on PEMS08 using multiple random seeds.

As shown in Table 3, removing individual components generally increases forecasting errors, although the effect is not uniform across all datasets and metrics. The variants w/o Freq Decomp and w/o Spectral Gate show relatively consistent degradation, suggesting that frequency-domain separation and adaptive spectral modulation contribute to the overall forecasting performance. Removing the Inception or gated modules also increases the errors in most settings. However, several variants obtain comparable or slightly better values on individual metrics. For example, on PEMS07, w/o Gated and w/o Adaptive Graph achieve marginally lower MAPE, while the MAE difference between w/o Adaptive Graph and the full model is only 0.01. We therefore do not interpret these small differences as evidence that every component consistently improves every metric. Instead, the results indicate that the effects of some spatial and gating components are dataset- and metric-dependent, while the complete model provides a competitive overall balance across the evaluated metrics.

We further examine the hypergraph’s design from two aspects. First, we vary the representation used for dynamic hyperedge generation, where Raw-HG, High-HG, and Low-HG construct hyperedges from mixed, high-frequency, and low-frequency representations, respectively. Second, while keeping Low-HG as the topology source, we compare the simplified propagation used in FEDHNet with a conventional degree-normalized variant. The results are summarized in Table 4. For the topology source, Low-HG obtains lower MAE, RMSE, and MAPE than Raw-HG on PEMS04. On PEMS07, Low-HG achieves lower MAE and MAPE, while its RMSE is very close to that of Raw-HG (32.98 versus 32.93). High-HG generally performs worse than Low-HG, particularly on PEMS07. On PEMS08, however, the fixed-seed differences between Raw-HG and Low-HG are marginal, with only a 0.02 difference in MAE. These results suggest that the effect of topology-source selection varies across datasets and metrics rather than showing a uniform advantage for a particular representation source. To further examine the marginal difference on PEMS08, we repeated Raw-HG and Low-HG using three random seeds (157, 42, and 2026). Raw-HG obtains 13.53 ± 0.05 MAE, 23.32 ± 0.04 RMSE, and 8.81 ± 0.03 MAPE, whereas Low-HG obtains 13.64 ± 0.17 MAE, 23.44 ± 0.30 RMSE, and 8.91 ± 0.13 MAPE. The 0.02 MAE advantage observed for Low-HG in the fixed-seed experiment is therefore not preserved across different random initializations. We regard this small fixed-seed difference as a marginal fluctuation rather than evidence of a stable advantage. Overall, low-frequency-guided hypergraph construction shows clearer benefits on PEMS04 and on MAE and MAPE for PEMS07, while its advantage is less evident on PEMS08. For the propagation scheme, neither formulation consistently dominates across all metrics. The simplified propagation achieves lower MAE on PEMS04, PEMS07, and PEMS08 and lower RMSE on PEMS04, whereas the normalized variant obtains slightly lower MAPE on PEMS04 and lower RMSE and MAPE on PEMS08. These results indicate that explicit degree normalization does not provide a consistent advantage for the learned soft hypergraph. We therefore retain the simplified formulation because it provides competitive performance without additional degree-normalization operations.

### 5.6. Sensitivity Analysis of High-Frequency Residuals

To examine how high-frequency residuals affect prediction, we conduct a sensitivity analysis by perturbing the high-frequency component during inference. A scaling factor α∈[0.0, 2.0] is introduced to scale the decoupled high-frequency representation $X_{\mathrm{high}}^{\left(\mathrm{out}\right)}$ before residual gated fusion:

(19) $$X_{\mathrm{fused}}=X_{\mathrm{low}}^{\left(\mathrm{out}\right)}+G_{\mathrm{fuse}}⊙\left(\alpha \cdot X_{\mathrm{high}}^{\left(\mathrm{out}\right)}\right)$$

The forecasting performance is then evaluated under different values of α on PEMS04 and PEMS08.

**Figure 5:**
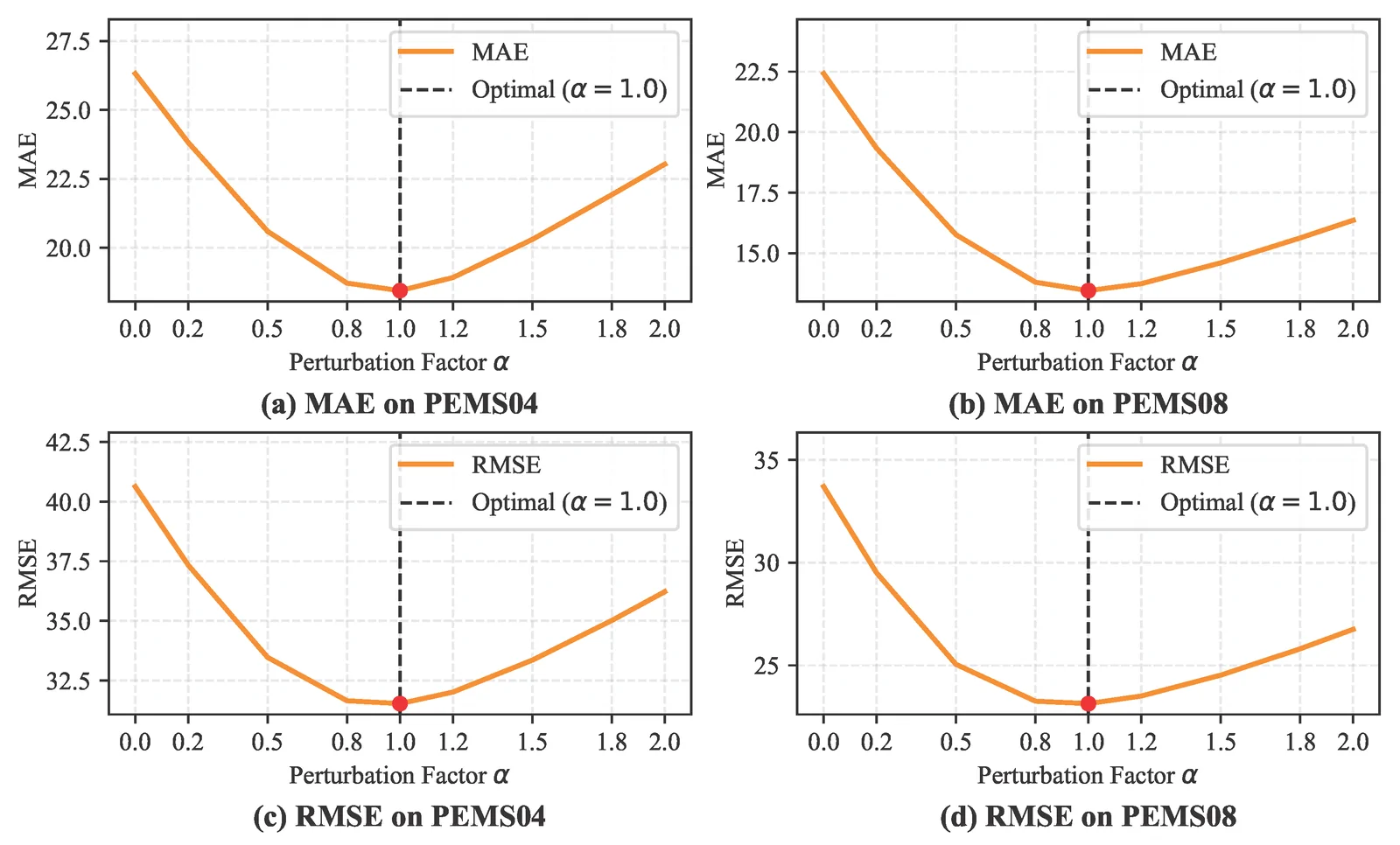
Sensitivity analysis of forecasting performance (MAE and RMSE) to high-frequency perturbations on the PEMS04 and PEMS08 datasets. The red dots indicate the original unscaled setting (α=1.0).

As shown in Figure 5, both MAE and RMSE exhibit a U-shaped trend. The best performance is achieved around α=1.0, indicating that the learned fusion module effectively balances the low-frequency latent representation and the high-frequency residual component. When α<1.0, the contribution of the high-frequency residual component is reduced, which is associated with increased prediction errors. When α>1.0, excessive amplification of the high-frequency component also degrades performance. These observations support the usefulness of controlled high-frequency residual information in the fusion process.

### 5.7. Interpretability of Dynamic Hypergraph Evolution

**Figure 6:**
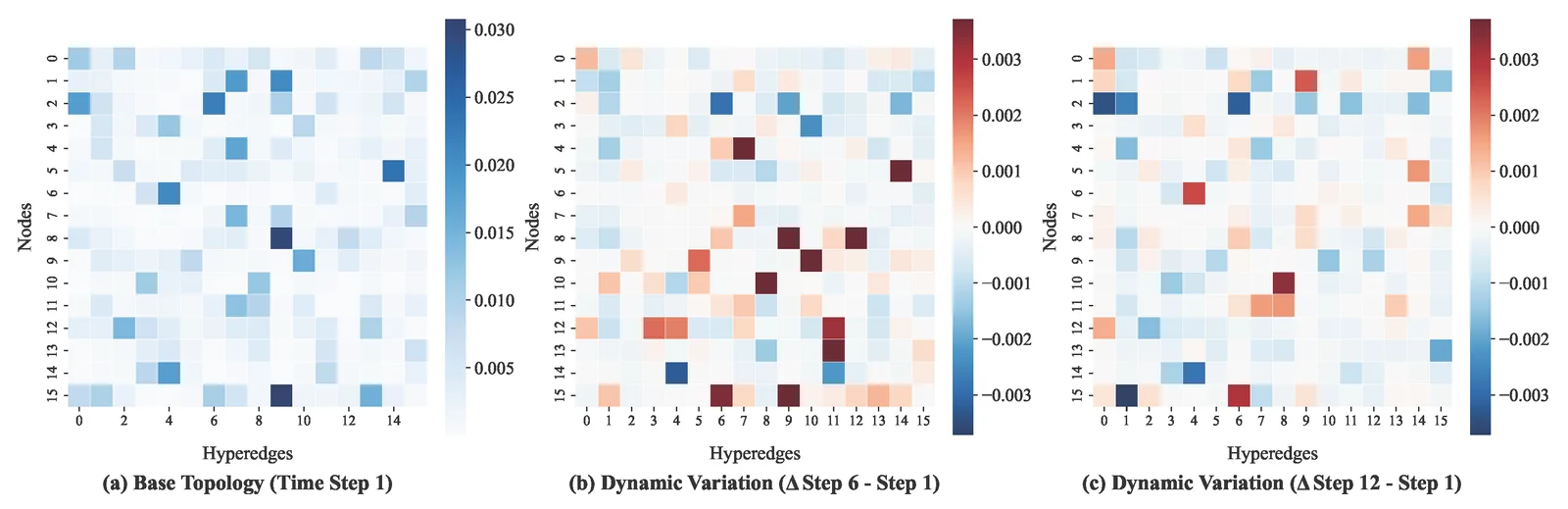
Visualization of the dynamic hyperedge incidence matrix at different temporal positions within the historical input window.

To visualize the temporal evolution of the learned high-order topology within the historical input window, Figure 6 presents the dynamic hyperedge incidence matrices at selected time steps. The first subfigure shows the incidence matrix at Time Step 1, while the remaining heatmaps show differences at Time Steps 6 and 12 relative to the initial state. The base incidence matrix exhibits non-uniform and concentrated node–hyperedge membership patterns, while the difference maps reveal localized changes over time. These observations indicate that the learned high-order structure evolves with the latent input while retaining substantial overlap with earlier assignments.

### 5.8. Multi-Horizon Forecasting Analysis

**Figure 7:**
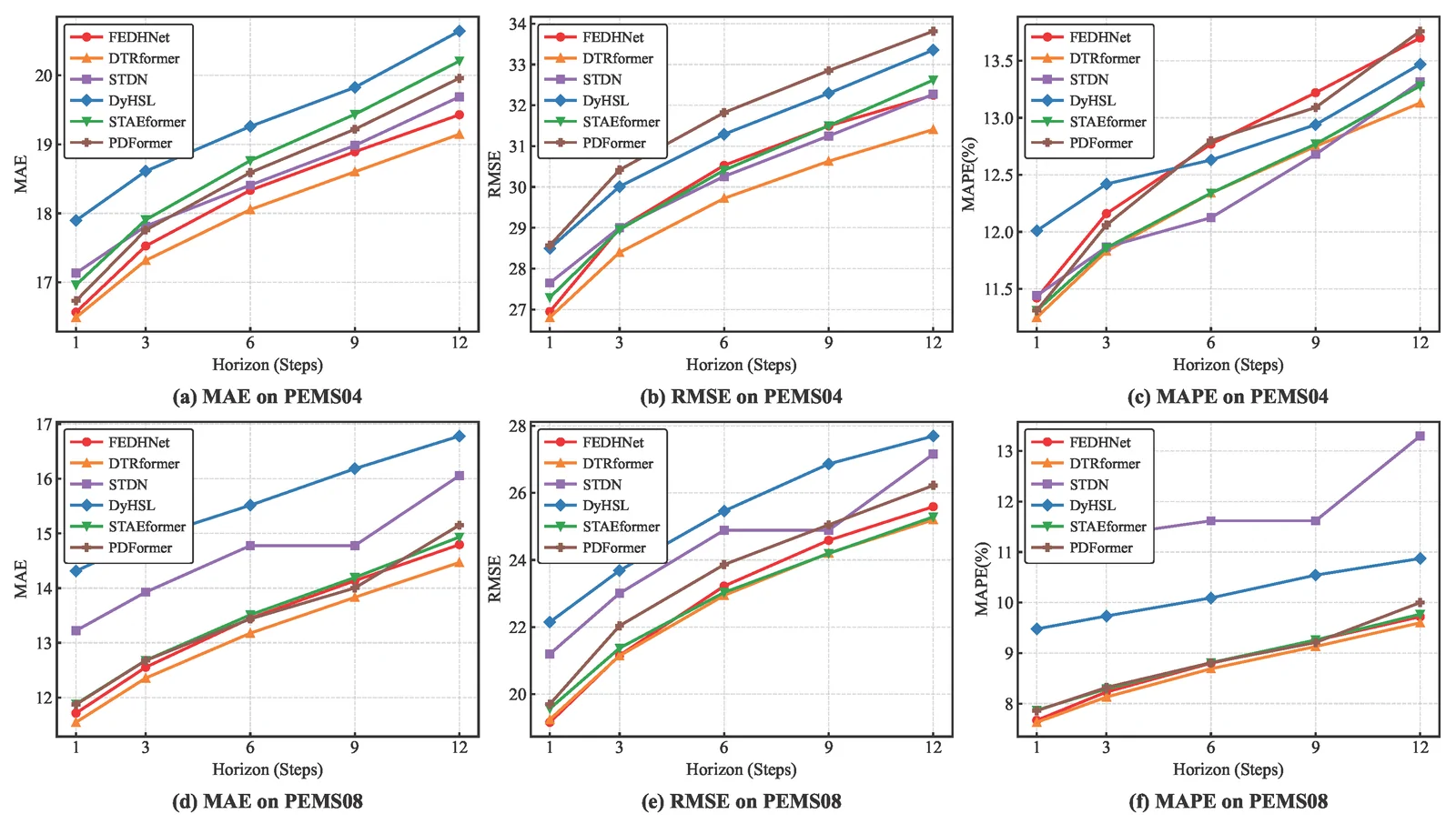
Multi-horizon forecasting errors at 5, 15, 30, 45, and 60 min on the PEMS04 and PEMS08 datasets.

To examine how forecasting performance changes with the prediction horizon, Figure 7 compares MAE, RMSE, and MAPE on PEMS04 and PEMS08 at representative forecasting horizons. Forecasting errors generally increase as the prediction horizon becomes longer. DTRformer achieves lower absolute errors in many settings, while FEDHNet remains competitive across the evaluated horizons. Compared with the closely related STDN and DyHSL baselines, FEDHNet generally achieves lower MAE and RMSE on both datasets, whereas the relative MAPE performance varies across datasets and forecasting horizons. These results indicate that FEDHNet maintains competitive multi-horizon forecasting performance rather than showing a uniform advantage at every prediction horizon.

### 5.9. Case Study: Visualizing Complex Traffic Dynamics

We further examine the temporal modeling ability of FEDHNet by comparing 60 min ahead forecasting results with the ground truth on PEMS04 nodes 10 and 150 over a 24 h period.

**Figure 8:**
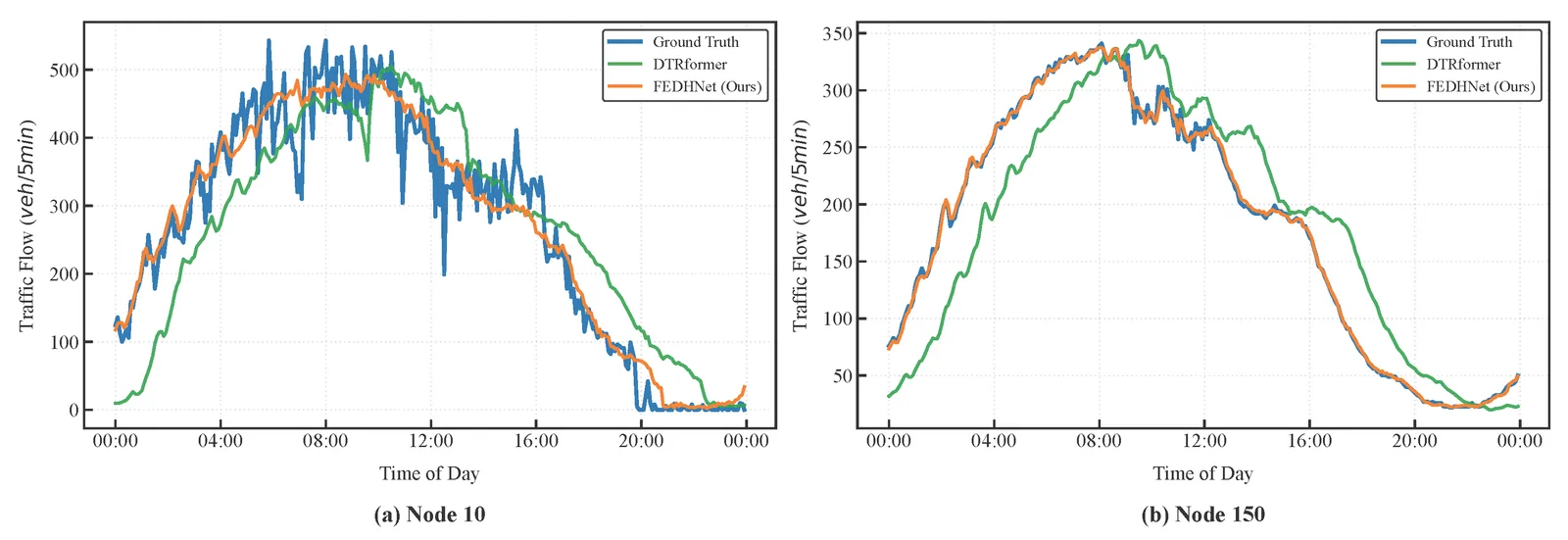
Visualization of 24 h traffic flow forecasting (60 min ahead) on the PEMS04 dataset.

As shown in Figure 8a, Node 10 contains sharp peaks and abrupt drops. In this example, FEDHNet follows several rapid traffic changes more closely than DTRformer, which is consistent with the intended role of the high-frequency branch in preserving rapidly varying information. Figure 8b shows the traffic flow of Node 150, which contains a prolonged peak and noticeable sensor noise. DTRformer tends to underestimate the peak volume, whereas FEDHNet better recovers the macroscopic traffic trend. The smoother prediction around noisy observations is also consistent with the intended denoising role of the gated high-frequency branch.

### 5.10. Hyperparameter Sensitivity Analysis

**Figure 9:**
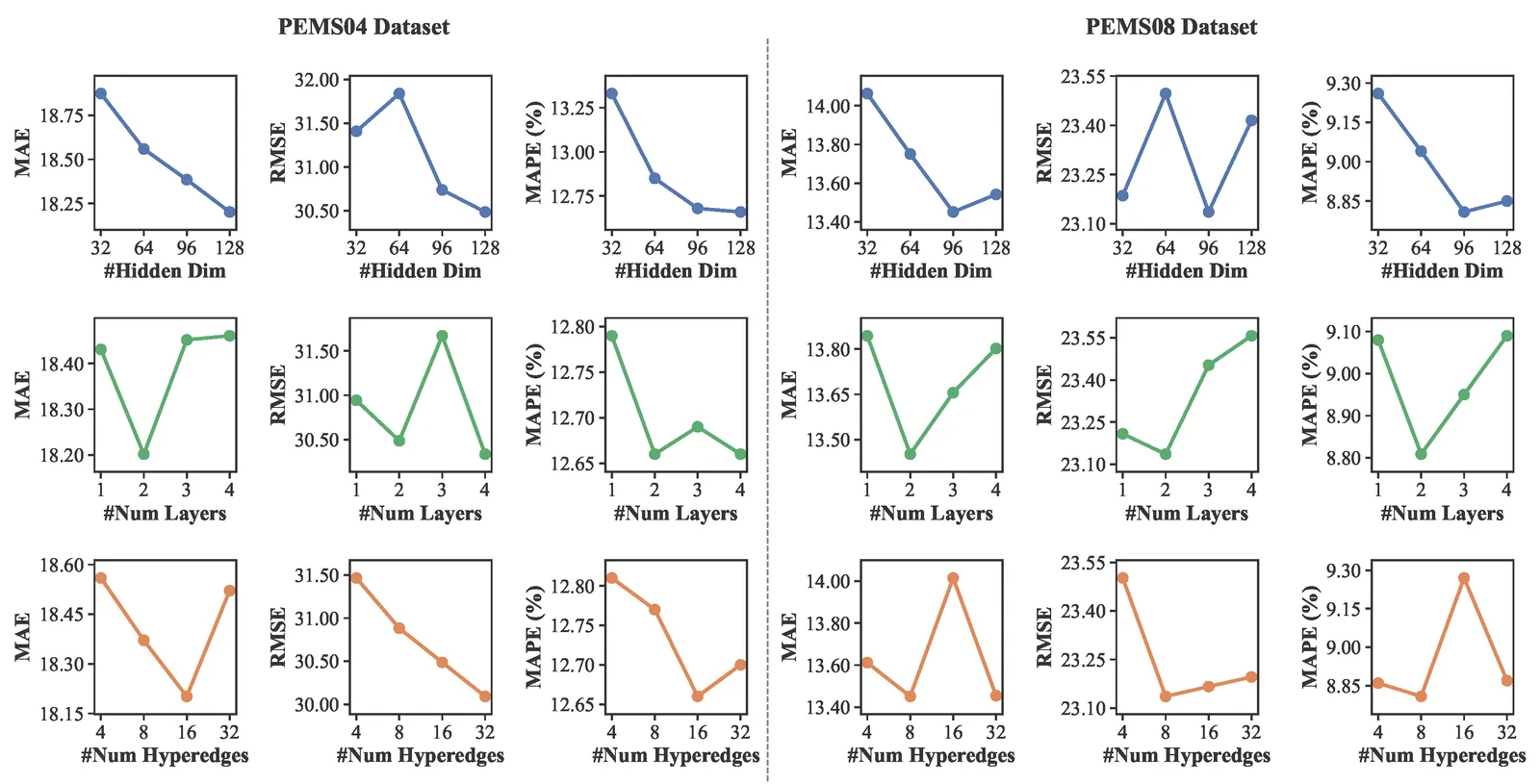
Hyperparameter sensitivity analysis of FEDHNet on PEMS04 and PEMS08, averaged over all 12 forecasting horizons.

We analyze three key hyperparameters of FEDHNet: the hidden dimension *D*, the number of network layers *L*, and the number of hyperedges *K*. Figure 9 reports the average performance over all 12 forecasting horizons on PEMS04 and PEMS08. On PEMS04, D=128 achieves the best results across all three metrics. For *L* and *K*, the adopted settings, L=2 and K=16, achieve lower MAE and MAPE, while L=4 and K=32 yield slightly lower RMSE. On PEMS08, D=96, L=2, and K=8 provide the best overall performance. These results show that the selected configurations provide a favorable overall performance across the evaluated metrics.

### 5.11. Sensitivity to Inference Batch Size

To examine the batch dependence of the dominant-period voting mechanism, we evaluate the same trained FEDHNet checkpoint on PEMS08 using inference batch sizes of 8, 16, 32, and 64. All 3549 test samples are evaluated exactly once without padding. As shown in Table 5, MAE, RMSE, and MAPE remain identical to four decimal places across all settings, and every mini-batch yields the same folding period P=2. These results indicate that the voting mechanism shows no observable sensitivity to inference batch size under the evaluated setting, although its dependence on batch composition remains in principle.

### 5.12. Computational Efficiency and Complexity Analysis

All efficiency results are measured on the same GPU under the same batch size. Table 6 compares FEDHNet with STAEformer and DTRformer on PEMS04 and PEMS08. The evaluation metrics include the total number of parameters, average training time per epoch, and total inference time on the test set. Although FEDHNet has a relatively large parameter size, it achieves lower training and inference time than the compared Transformer-based baselines under the same experimental environment. On PEMS04, FEDHNet requires 11.60 s per training epoch, which is a 71.6% reduction compared with DTRformer’s 40.80 s. Its inference time is also reduced to 1.75 s, outperforming both STAEformer and DTRformer. Similar efficiency gains are observed on PEMS08.

We further analyze the computational characteristics of FEDHNet. The FFT-based adaptive spectral decomposition has a complexity of O(NDTlogT), and the 2D Inception-based high-frequency branch can be efficiently parallelized over folded temporal grids. The dynamic hypergraph learning branch operates on a node–hyperedge incidence tensor, with complexity O(TNKD) for node–hyperedge aggregation and broadcasting. Although FEDHNet still uses a lightweight temporal Transformer after feature fusion, the frequency-guided routing reduces the burden of directly modeling entangled traffic patterns with heavy global attention. Therefore, the empirical efficiency gain mainly comes from parallel spectral decomposition, compact hypergraph aggregation, and lightweight high-frequency convolution rather than from completely removing attention operations.

## 6. Conclusions

In this paper, we proposed FEDHNet, a Frequency-Guided Dynamic Hypergraph Network for traffic flow forecasting. The main idea is to use adaptive spectral decomposition not only to separate heterogeneous latent components but also to provide a smoother representation basis for dynamic high-order topology learning. By constructing dynamic hyperedges from low-frequency latent representations, FEDHNet reduces the direct influence of rapidly varying latent components on hyperedge generation and high-order topology inference. Meanwhile, the high-frequency branch uses a lightweight 2D Inception module with GLU-based gated modulation to capture complementary rapidly varying information. A low-frequency-anchored residual fusion module then adaptively integrates the high-frequency residual component for multi-step prediction. Experiments on four public PeMS datasets show that FEDHNet achieves competitive forecasting accuracy and multi-horizon performance, together with favorable computational efficiency compared with recent spatiotemporal forecasting baselines. The ablation and sensitivity analyses generally support the roles of frequency decomposition, dynamic hypergraph learning, and high-frequency residual modeling components, while also showing that their effects can vary across datasets and metrics. The short-term forecasts produced by FEDHNet can support practical ITS applications such as traffic monitoring, congestion warning, and route guidance. Despite these advantages, FEDHNet still has several limitations. First, the current spectral decomposition is performed within a fixed historical window, which may limit its ability to capture longer-term periodic patterns. Second, external factors such as weather, accidents, events, and road control policies are not explicitly modeled. Third, although FEDHNet provides a favorable accuracy–efficiency trade-off, its absolute prediction accuracy is not uniformly superior to the strongest Transformer-based baselines on all datasets. In future work, we will incorporate external spatiotemporal contexts and explore more adaptive multi-resolution frequency-guided topology learning mechanisms.

## Figures and Tables

**Table 1:** Statistical description of the experimental datasets.

Dataset	Nodes	Edges	Steps	Time Span	Features
PEMS03	358	547	26,208	1 September 2018–30 November 2018	1
PEMS04	307	340	16,992	1 January 2018–28 February 2018	3
PEMS07	883	866	28,224	1 May 2017–31 August 2017	1
PEMS08	170	295	17,856	1 July 2016–31 August 2016	3

**Table 2:** Forecasting performance comparison of different models averaged over all 12 forecasting horizons on the PEMS03, PEMS04, PEMS07, and PEMS08 datasets. The best results are highlighted in **bold**, and the second-best results are underlined.

Model	PEMS03			PEMS04			PEMS07			PEMS08		
	MAE	RMSE	MAPE (%)	MAE	RMSE	MAPE (%)	MAE	RMSE	MAPE (%)	MAE	RMSE	MAPE (%)
ARIMA	35.41	47.59	33.78	33.73	48.80	24.18	38.17	59.27	19.46	31.09	44.32	22.73
STGCN	17.55	30.42	17.34	21.16	34.89	13.83	25.33	39.34	11.21	17.50	27.09	11.29
DCRNN	17.99	30.31	18.34	21.22	33.44	14.17	25.22	38.61	11.82	16.82	26.36	10.92
Graph WaveNet	19.12	32.77	18.89	24.89	39.66	17.29	26.39	41.50	11.97	18.28	30.05	12.15
ASTGCN	17.34	29.56	17.21	22.93	35.22	16.56	24.01	37.87	10.73	18.25	28.06	11.64
STSGCN	17.48	29.21	16.78	21.19	33.65	13.90	24.26	39.03	10.21	17.13	26.80	10.96
Z-GCNETs	16.64	28.15	16.39	19.50	31.61	12.78	21.77	35.17	9.25	15.76	25.11	10.01
STGODE	16.50	27.84	16.69	20.84	32.84	13.77	22.99	37.54	10.14	16.81	25.97	10.62
STG-NCDE	15.71	27.08	**13.28**	19.29	31.16	12.73	21.12	34.02	9.12	16.60	26.08	10.81
DyHSL	15.66	27.49	15.67	19.32	31.32	12.72	20.73	34.69	8.67	15.61	25.47	10.19
PDFormer	14.94	25.39	15.82	18.57	31.78	12.68	19.83	32.87	8.53	13.50	23.75	8.89
STAEformer	15.46	26.83	15.54	18.78	30.42	12.38	19.49	33.10	8.33	13.54	23.00	8.85
STPGNN	**14.68**	**24.14**	15.35	22.90	35.42	15.87	20.22	33.11	8.83	14.92	23.88	9.90
STDN	15.43	27.20	16.09	18.49	30.30	**12.32**	20.38	33.46	9.12	14.79	24.84	11.98
DTRformer	14.76	25.63	15.14	**18.03**	**29.65**	12.34	**19.19**	**32.46**	**8.04**	**13.19**	**22.90**	**8.71**
**FEDHNet (Ours)**	15.11	24.33	15.89	18.20	30.36	12.66	19.27	32.98	8.15	13.45	23.14	8.81

**Table 3:** Component-wise ablation study of FEDHNet on the PEMS04, PEMS08, and PEMS07 datasets averaged over all 12 forecasting horizons.

Variant	PEMS04			PEMS08			PEMS07		
	MAE	RMSE	MAPE (%)	MAE	RMSE	MAPE (%)	MAE	RMSE	MAPE (%)
w/o Freq Decomp	18.47	30.53	12.77	13.53	23.25	8.94	19.34	33.25	8.20
w/o Spectral Gate	18.75	31.78	13.20	13.68	23.48	8.90	19.54	33.79	8.23
w/o Hypergraph	18.38	30.28	12.66	13.45	23.25	8.85	19.31	32.75	8.18
w/o 2D Inception	18.51	30.47	13.25	14.40	23.41	9.41	19.32	32.96	8.22
w/o Gated	18.48	30.38	13.29	13.56	23.38	8.87	19.28	32.97	8.11
w/o Adaptive Graph	18.57	32.06	12.74	13.65	23.43	8.89	19.26	32.99	8.11
FEDHNet (Full)	18.20	30.36	12.66	13.45	23.14	8.81	19.27	32.98	8.15

**Table 4:** Hypergraph design ablation on PEMS04, PEMS07, and PEMS08 averaged over all 12 forecasting horizons.

Variant	PEMS04			PEMS07			PEMS08		
	MAE	RMSE	MAPE (%)	MAE	RMSE	MAPE (%)	MAE	RMSE	MAPE (%)
Raw-HG	18.65	31.57	12.72	19.73	32.93	8.50	13.47	23.29	8.83
High-HG	18.36	30.72	12.92	20.13	33.33	8.73	13.73	23.21	9.06
Low-HG + Normalized	18.35	31.27	12.59	19.54	33.22	8.19	13.54	23.11	8.76
Low-HG + Simplified (FEDHNet)	18.20	30.36	12.66	19.27	32.98	8.15	13.45	23.14	8.81

**Table 5:** Sensitivity of FEDHNet to different inference batch sizes on PEMS08.

Batch Size	MAE	RMSE	MAPE (%)	Folding Period
8	13.4517	23.1362	8.8085	2
16	13.4517	23.1362	8.8085	2
32	13.4517	23.1362	8.8085	2
64	13.4517	23.1362	8.8085	2

**Table 6:** Computational efficiency comparison on the PEMS04 and PEMS08 datasets. The best time efficiency results are highlighted in bold.

Model	PEMS04			PEMS08		
	Params	Train (s/ep)	Infer (s)	Params	Train (s/ep)	Infer (s)
STAEformer	1.35 M	29.80	3.22	1.22 M	14.90	1.46
DTRformer	2.51 M	40.80	3.97	2.33 M	31.50	2.26
**FEDHNet (Ours)**	4.25 M	**11.60**	**1.75**	2.11 M	**7.60**	**0.97**

## Data Availability

The datasets used in this study are publicly available from the Caltrans Performance Measurement System (PeMS).
